# Supplementary material for: Feasibility of tobacco cessation intervention at non-communicable diseases clinics: A qualitative study from a North Indian State
Source: PLoS One. 2023 May 4;18(5):e0284920. doi: 10.1371/journal.pone.0284920 (PMC10159160; doi:10.1371/journal.pone.0284920)
Supplement: Attachment — Submitted filename: response to reviewers.docx [file pone.0284920.s001.docx]

**Response sheet**

| **Reviewer no.** | **Comment from the reviewer** | **Response by the authors** | **Page no & line no** |
| --- | --- | --- | --- |
| **Journal Requirements:** |  | We thank the Editor, and the reviewers for their valuable comments. |  |
|  | When submitting your revision, we need you to address these additional requirements.  1. Please ensure that your manuscript meets PLOS ONE's style requirements, including those for file naming. The PLOS ONE style templates can be found at  https://journals.plos.org/plosone/s/file?id=wjVg/PLOSOne_formatting_sample_main_body.pdf and  <https://journals.plos.org/plosone/s/file?id=ba62/PLOSOne_formatting_sample_title_authors_affiliations.pdf> | We thank the journal for the suggestion.  We have followed the journal’s style requirements including those for file naming in the revised uploads. | **-** |
|  | 2. Please provide additional details regarding participant consent. In the ethics statement in the Methods and online submission information, please ensure that you have specified whether the ethics committee approved the verbal/oral consent procedure. | The Institute Ethics Committee (IEC), Post Graduate Institute of Medical Education & Research (PGIMER), Chandigarh, India (IEC no. INT/IEC/2017/1361) granted ethical approval for the study. Due permissions were sought prior from the State Tobacco Control Cell & NCD Control Cell, Department of Health & Family Welfare, Government of Punjab. India. All respondents were apprised about the study, and signed informed consent was obtained whenever there was a physical interview.For interviews conducted telephonically, the participant's consent was taken verbally during the interview. They were sent a consent form and participant information sheet via email address and were requested to share a signed and scanned copy of the same with us.The researcher assured the study respondents about their anonymity and informed them that their identities would not be disclosed in the aggregate data findings. | **-** |
|  | 3. We note that you have indicated that data from this study are available upon request. PLOS only allows data to be available upon request if there are legal or ethical restrictions on sharing data publicly. For more information on unacceptable data access restrictions, please see http://journals.plos.org/plosone/s/data-availability#loc-unacceptable-data-access-restrictions. | The data are not publicly available because the data is collected from a small group of participants which may risk the identification of respondents and could compromise the privacy of research participants. Further, the study presents the data analyzed as part of qualitative research, and we have added excerpts of the transcripts within the manuscript that are relevant to the study. However, the data that support the findings of this study are available from the corresponding author, [SG], upon reasonable request. | **-** |
|  | In your revised cover letter, please address the following prompts:  a) If there are ethical or legal restrictions on sharing a de-identified data set, please explain them in detail (e.g., data contain potentially sensitive information, data are owned by a third-party organization, etc.) and who has imposed them (e.g., an ethics committee). Please also provide contact information for a data access committee, ethics committee, or other institutional body to which data requests may be sent.  b) If there are no restrictions, please upload the minimal anonymized data set necessary to replicate your study findings as either Supporting Information files or to a stable, public repository and provide us with the relevant URLs, DOIs, or accession numbers. For a list of acceptable repositories, please see http://journals.plos.org/plosone/s/data-availability#loc-recommended-repositories. We will update your Data Availability statement on your behalf to reflect the information you provide. | We have addressed the both the prompts a & b in the revised cover letter. | **-** |
|  | 4. Please upload a new copy of Figures 1 and 3 as the detail is not clear. Please follow the link for more information: https://blogs.plos.org/plos/2019/06/looking-good-tips-for-creating-your-plos-figures-graphics/ https://blogs.plos.org/plos/2019/06/looking-good-tips-for-creating-your-plos-figures-graphics/ | We thank the journal for the comment.  We have uploaded new copy of figures 1 and 3 with enhanced clarity and readability on the journal submission portal. | **-** |
|  | 5. We note you have included a table to which you do not refer in the text of your manuscript. Please ensure that you refer to Table 1 in your text; if accepted, production will need this reference to link the reader to the Table. | We thank the journal for the suggestion. We have included Table-1 in the text of the manuscript. | **Page no: 11**  **Line no:234** |
|  | 6. Please review your reference list to ensure that it is complete and correct. If you have cited papers that have been retracted, please include the rationale for doing so in the manuscript text, or remove these references and replace them with relevant current references. Any changes to the reference list should be mentioned in the rebuttal letter that accompanies your revised manuscript. If you need to cite a retracted article, indicate the article’s retracted status in the References list and also include a citation and full reference for the retraction notice. | We have reviewed the reference list for completeness and correctness and updated the list. | **Page no:37 -42**  **Line no:834-1062** |
| **Reviewer 1** | Background: Inclusion of data on “tobacco use disorders” whilst interesting, is a bit confusing as the term is not explained and it is not clear how these disorders might play a role in cessation, NCDs and whether they were tackled specifically in this feasibility assessment. Suggest rewording for clarity or removing the sentence. “The World Health Assembly affirmed a 30 percent relative reduction in current tobacco usage (daily and occasional) between 2010 and 2025 among individuals aged 15 years and above” Needs citation. | We thank the reviewer for the comment. We have removed this sentence from the revised manuscript as suggested.  Also, we have added the citation for the suggested statement in the revised manuscript. | **Page no:4**  **Line no:83 - 85** |
|  | Methods: Typo on the 4th line. “13.4t”? | We thank the reviewer for the comment. We have corrected the typo in the methods section. “According to the Global Adult Tobacco Survey-2 (2016-2017), the state's current tobacco use prevalence is 13.4 %”. | **Page no:6**  **Line no:132-134** |
|  | Strengths and Limitations: “Second, the results may be subject to responder bias; since all participants were from the public sector, there is a tendency to present a less realistic view”. Could you clarify what is meant by “less realistic view”. Do you mean public sector workers are overly idealistic, or that their perspective is simply limited, or…? | We are grateful to the reviewer for the comment. Here we simply mean that since all our study respondents were from public sector settings; this makes the perspective limited and the viewpoint of health care providers from the private sector was missed. We have revised the text in the manuscript. | **Page no: 35**  **Line no:797-800** |
| **Reviewer 2** | As an early-career researcher in this area, I would like to express my compliment to the authors who conducted this study as I believe this article might enrich the body of knowledge in tobacco cessation work as valuable evidence. I do agree that integrating tobacco cessation service package into regular healthcare service could be beneficial to encourage people quit smoking. However, there is limited evidence regarding this matter from developing countries. Regarding the manuscript, I have several points I would like to elaborate below and hopefully could be an advantageous comment toward your work. | We thank the reviewer for the encouraging words, | **-** |
|  | 1. In general, I found some mistyped words, redundant parts, and uncommon academic English words. I also found many uncommon vocabularies that might be unfamiliar for public, namely ASHA workers, Panchayati Raj Institution, Ayuverdic, OTT, etc. You may consider to give footnote or brief explanation of the words. Moreover, through the manuscript, I found many acronyms and sometimes it became obstacle to follow the content. | We thank the reviewer for the comment.  We have added brief explanation of these terms as footnotes and expanded the acronyms in the revised manuscript as suggested by the reviewer. | **Page no:15,19,24, 25, 26**  **Line no: 320-321, 443-445, 532-540, 566-568, 591-592,** |
|  | 2. For the title, I would suggest to consider cut it shorter. How about this title? "The Feasibility of Tobacco Cessation Intervention at Non-communicable Diseases Clinics: A Qualitative Study From North Indian State". | We thank the reviewer for the valuable suggestion. We have revised the title as suggested by the reviewer. | **Page no: 1**  **Line no:3-4** |
|  | 3. In the abstract, I would suggest to paraphrase or state the objective of the study clearly. For the background part in the abstract, I also think this part is not strong enough to catch reader's attention. Moreover, I would consider the first sentence in the conclusion part to be deleted. | We are grateful to the reviewer for the comment. We have revised the text in the background part and removed the first sentence in the conclusion part as suggested by the reviewer. | **Page no:1, 2**  **Line no:8-14, 39-42** |
|  | 4. In the main manuscript, of Background section, I would suggest a more concise background. In general, background section should consist of rationale, what's known, what's unknown, and the significancy and objective of the study. The background could elaborate on the NCD in India, how tobacco cessation is needed but scarce (for example, depends on the situation in the India), what's previous studies say about the integration program of NCD clinics and what's the gap of the study in current situation, the significancy of the study and study's objective. In detail, paragraph 1 explained the high prevalence of NCDs and tobacco consumption in India. I would rather stick to the "one paragraph, one main idea". Moreover, I believe the 2-4 paragraphs could be summarised into shorter one with the idea of tobacco cessation service and its demand and regional data regarding this case. There are many global data stated in the body of paragraph, however I believe regional or national data might be useful to support the notion. | We thank the reviewer for the comment. We have revised the text as per the format suggested by the reviewer in the revised manuscript.  We have also highlighted the gap and significance of the current study as suggested by the reviewer.  Also, we have curtailed the global data from the manuscript as suggested by the reviewer in the revised manuscript. | **Page no:3,4, 5, 6**  **Line no:66-79, 80-89, 98-103, 107-124** |
|  | 5. In the Method section, I am not sure what is the meaning of this data "13,4t"? Regarding the GATS, is the latest available? I also not quite sure whether the subjects of the study came from same hospitals/working places? If yes, I think it would be great if the authors could mention that and including the matter in the table of analysis. | We thank the reviewer for the comment. We have corrected the typo in the methods section. “According to the Global Adult Tobacco Survey-2 (2016-2017), the state's current tobacco use prevalence is 13.4 %”.  The last GATS data available for India is of GATS-round two, 2016-2017.  The study subjects were from various health facilities across different districts of Punjab. | **Page no:6**  **Line no:132-134** |
|  | 6. In the Result section, as I read through the manuscript, sometime it is difficult to differentiate the result from each theme. For example, in the second theme of demand, I am not expecting the discussion would address the resistance or hesitation from patient side. Due to this matter, I would suggest to give additional information in the Method section regarding the explanation or meaning of each theme. | We thank the reviewer for the comment. We have added a brief about each theme in the methods section as suggested by the reviewer in the revised manuscript. | **Page no:9**  **Line no:197-204** |
|  | 7. In the Discussion section, I would suggest the first paragraph is not necessary. Otherwise, you may consider to summarise the objective of the study and highlight of the key findings from the Result section. The following paragraphs may discuss each key findings and comparing the current study's findings to the previous studies. Reasonings or rationalization of the trend, either current study and previous studies are supporting or against each other, may be added, as well. Moreover, in the sub-section of strength and limitation, the authors mentioned "rigorous qualitative analysis (P.39), you may want to add how rigorous or what analysis or measurements had been done to make this study is rigorous. Overall, I would suggest sub-section of the implication and recommendation is not necessary since the content is a summary of discussion and restate again in the conclusion. | We are grateful to the reviewer for the comment. We have removed the first para as suggested by the reviewer from the discussions section. We have restructured and revised the text as suggested by the reviewer.  We have added the analysis measurements and rigor section to the revised manuscript as suggested.  We have also curtailed the sub section of implications and recommendations and revised the conclusion section. | **Page no:26, 28,29,31-32,33,34**  **Line no:594-600, 619-621, 626-629, 633-636,651-655,669-673,707-709,717-720,725-730,740-744,754-756,761-763, 779-781, 791-794** |
|  | 8. Lastly. I found some minor errors in Reference section. There is inconsistency in reference style, for example in addressing World Health Organization (WHO), and I am not sure about the reference number 25 and 30. You may consider to revise it if needed. | We thank the reviewer for the comment.  We have reviewed the references and updated the suggested. | **Page no:37 -42**  **Line no:834-1062** |
| The authors feel that the manuscript has been immensely improved after incorporating the comments of editorial board, and we thank the board for their time and efforts. | | | |
