# Supplementary material for: Feasibility of tobacco cessation intervention at non-communicable diseases clinics: A qualitative study from a North Indian State
Source: PLoS One. 2023 May 4;18(5):e0284920. doi: 10.1371/journal.pone.0284920 (PMC10159160; doi:10.1371/journal.pone.0284920)
Supplement: Attachment — Submitted filename: Plos One_review comments.docx [file pone.0284920.s002.docx]

**Manuscript Title:** Feasibility of Tobacco Cessation Intervention at Non-communicable Diseases Clinics: A Qualitative Study from a North Indian State

| **Section** | **Manuscript Line item** | **Comment(s)/Suggestion(s)** |
| --- | --- | --- |
| **Overall** |  |  |
|  | Background:  Line 10-14: repetitive words in both sentences | merge and make one sentence |
| **Abstract** | Results: The respondent's Mean ± SD age was 39.2± 9.2 | Add “years” after 9.2 |
|  | Methods: Line 16-18 | To be removed or rephrased, as this intervention was not part of this manuscript  Also, this intervention should be cited in the methodology section  Year of study |
|  | SOPs | Expand at the first mention |
|  | Order of Authors | Remove MD |
| **Short title** |  | Rephrase and make it shorter |
| **INTRODUCTION** | Line 115-122: repetitive words in both sentences | merge and make one sentence |
| **METHODS** |  | The intervention should be cited in the methodology section |
