# Supplementary material for: Feasibility of tobacco cessation intervention at non-communicable diseases clinics: A qualitative study from a North Indian State
Source: PLoS One. 2023 May 4;18(5):e0284920. doi: 10.1371/journal.pone.0284920 (PMC10159160; doi:10.1371/journal.pone.0284920)
Supplement: Attachment — Submitted filename: Reviewers comments.docx [file pone.0284920.s003.docx]

Reviewer’s comments:

Abstract

1. Units of measurement not mentioned “respondent's Mean ± SD age was 39.2± 9.2,” page 1, line no. 24
2. Results seem to be part of methodology, “the data was analysed……. for implementation, modifications required, and integrational perspectives). As these are the predefined things on which you will analyze the data and are part of analysis and not result a nd that too in middle of results.. In result section authors can presents the findings under these themes. page 1-2 line 25-30.

Manuscript:

1. Use updated data for latest NCD mortality, “2016” data seems to be too old. The 2019 data states NCD mortality to be around 66% while 2022. Page 3, line no 55-7
2. Correct the name of programme “such as Prevention and Control of Cancer, Diabetes, Cardiovascular Diseases, and Stroke (NPCDCS) page 4, line no. 91-2
3. Write abbreviation at first place where the full form has been used. “NTCP also emphasizes” Page 5, line no 96
4. Typographical error “Figure-1 health care system in India” need to be corrected as Health. Page 7, line no 148
5. Reframe the sentence,” The district hospital….geographical area and population” page 7-8, line no 162-64
6. Under subheading **“Study population & sampling”.** How many district hospitals out of total District hospitals were selected in your current study? Brief about the technique was used to select the study hospitals and the criteria used for selecting them. How many district hospitals were enrolled for selected 45participants for interview? Page 7-8, line no 160-169
7. Suggestion for changing “focused life history” to demographic details. Page 8, line no 183
8. Units of measurement not mentioned “respondent's Mean ± SD age was 39.2± 9.2,” page 10, line no. 230
9. Table 1: Since both mean and median are the measure of central tendencies, the author is suggested to select one out these two depending upon the distribution of data until and unless the author wants to conclude some inference using both of them. Page 11, line no 232
10. “The results are categorized into the key focus areas as preset domains for feasibility as suggested by Bowen et al. [31]” is a part of statistical analysis. Page 11, line no 237-38
11. “They also stressed the **promotion…”** Whom you are referring as “they” in this statement. Page 12, line no 268
12. Another limitation of study can be “surrogacy interviews” by HCP for barriers at patient level.
13. Manuscript editing suggested as at some places two words are joined together and at some places one word is bifurcated.
