# Supplementary material for: Feasibility of tobacco cessation intervention at non-communicable diseases clinics: A qualitative study from a North Indian State
Source: PLoS One. 2023 May 4;18(5):e0284920. doi: 10.1371/journal.pone.0284920 (PMC10159160; doi:10.1371/journal.pone.0284920)
Supplement: Attachment — Submitted filename: response to reviewers.docx [file pone.0284920.s004.docx]

**Response sheet**

| **Reviewer no.** | **Comment from the reviewer** | **Response by the authors** | **Page no & line no** |
| --- | --- | --- | --- |
|  |  | We thank the Editor and the reviewers for their valuable comments. | **-** |
| **Reviewer 4** | Abstract   1. Units of measurement not mentioned “respondent's Mean ± SD age was 39.2± 9.2,” page 1, line no. 24 | We thank the reviewer for the comment. We have added the units of measurement to the revised manuscript. | **Page no:2**  **Line no:24** |
|  | 2. Results seem to be part of methodology, “the data was analysed……. for implementation, modifications required, and integrational perspectives). As these are the predefined things on which you will analyze the data and are part of analysis and not result and that too in middle of results.. In result section authors can presents the findings under these themes. page 1-2 line 25-30. | We thank the reviewer for the suggestion. As suggested we have incorporated the predefined themes into the result section and removed them from the methods section in the revised manuscript. | **Page no:2**  **Line no:25-36** |
|  | Manuscript:   1. Use updated data for latest NCD mortality, “2016” data seems to be too old. The 2019 data states NCD mortality to be around 66% while 2022. Page 3, line no 55-7 | We are grateful to the reviewer for the suggestion. We have incorporated the latest WHO-NCD monitor 2022 data into the revised manuscript. | **Page no:3**  **Line no:54-55** |
|  | 2. Correct the name of programme “such as Prevention and Control of Cancer, Diabetes, Cardiovascular Diseases, and Stroke (NPCDCS) page 4, line no. 91-2 | We have revised the text as suggested by the reviewer. | **Page no:4**  **Line no:89** |
|  | 3. Write abbreviation at first place where the full form has been used. “NTCP also emphasizes” Page 5, line no 96 | We have incorporated the abbreviation along with the full form in the revised manuscript as suggested by the reviewer. | **Page no:4**  **Line no:88** |
|  | 4. Typographical error “Figure-1 health care system in India” need to be corrected as Health. Page 7, line no 148 | We thank the reviewer for the suggestion and we have corrected the typographical error in the revised manuscript. | **Page no:7**  **Line no:144** |
|  | 5. Reframe the sentence,” The district hospital….geographical area and population” page 7-8, line no 162-64 | We are grateful to the reviewer for the suggestion. We have reframed the sentence as suggested by the reviewer in the revised manuscript. | **Page no:7**  **Line no:158-159** |
|  | 6. Under subheading **“Study population & sampling”.** How many district hospitals out of total District hospitals were selected in your current study? Brief about the technique was used to select the study hospitals and the criteria used for selecting them. How many district hospitals were enrolled for selected 45participants for interview? Page 7-8, line no 160-169 | We thank the reviewer for the comment. Before the implementation of the intervention package, a state-level training workshop was conducted in collaboration with the State Tobacco Control Cell wherein all 22 districts of the state were requested to nominate their HCPs to attend the training. There was representation from each district hospital at the training. Later, we reached out to these participants across different districts ensuring representation from each district across the different categories of stakeholders interviewed. Forty-five participants (12 medical officers, 13 counselors, 10 nurses, and 10 program officers) were interviewed using purposive sampling from the cohort of trained HCPs. Following the Principle of Redundancy, the respondents under each category were interviewed until no new information emerged. We have added the text to the revised manuscript. | **Page no:8**  **Line no:162-170** |
|  | 7. Suggestion for changing “focused life history” to demographic details. Page 8, line no 183 | We thank the reviewer for the suggestion and we have revised the text as suggested in the revised manuscript. | **Page no:9**  **Line no:190-191** |
|  | 8. Units of measurement not mentioned “respondent's Mean ± SD age was 39.2± 9.2,” page 10, line no. 230 | We thank the reviewer for the comment. We have added the units of measurement to the revised manuscript. | **Page no:11**  **Line no:239** |
|  | 9. Table 1: Since both mean and median are the measure of central tendencies, the author is suggested to select one out these two depending upon the distribution of data until and unless the author wants to conclude some inference using both of them. Page 11, line no 232 | We thank the reviewer for the comment. In the revised manuscript, we have removed the mean and retained only the median (& IQR) in table 1 as the distribution is skewed and to understand the data’s center and spread. | **Page no:11**  **Line no:241-242** |
|  | 10. “The results are categorized into the key focus areas as preset domains for feasibility as suggested by Bowen et al. [31]” is a part of statistical analysis. Page 11, line no 237-38 | We thank the reviewer for the suggestion. We have shifted the text to the analysis section as suggested by the reviewer. | **Page no:10**  **Line no:219-220, 224-225** |
|  | 11. “They also stressed the **promotion…”** Whom you are referring as “they” in this statement. Page 12, line no 268 | ‘They’ refers to the medical officer's statement. We have revised the text in the manuscript. | **Page no:12**  **Line no:272** |
|  | 12. Another limitation of study can be “surrogacy interviews” by HCP for barriers at patient level | We thank the reviewer for the suggestion. We have incorporated the limitation into the revised manuscript. | **Page no:35**  **Line no:798-799** |
|  | 13. Manuscript editing suggested as at some places two words are joined together and at some places one word is bifurcated. | We thank the reviewer for the suggestion. We have edited the manuscript for spacing and formatting changes. | **-** |
| **Reviewer 3** | Background:  Line 10-14: repetitive words in both sentences  merge and make one sentence | We thank the reviewer for the suggestion. We have merged and rephrased the sentences in the revised manuscript. | **Page no:1**  **Line no:10-12** |
|  | **Abstract**  Results: The respondent's Mean ± SD age was 39.2± 9.2  Add “years” after 9.2 | We thank the reviewer for the comment. We have added the units of measurement to the revised manuscript. | **Page no:2**  **Line no:24** |
|  | Methods: Line 16-18  To be removed or rephrased, as this intervention was not part of this manuscript  Also, this intervention should be cited in the methodology section  Year of study  SOPs: Expand at the first mention  Order of Authors: Remove MD | We thank the reviewer for the suggestion. We have rephrased the text in the revised manuscript.  Also, we have added brief text and cited the intervention development methodology (published elsewhere), and the year of the study in the revised manuscript. We have also, expanded Standard Operating Procedures (SOPs) and removed MD from the order of authors as suggested by the reviewer in the revised manuscript. | **Page no:1,2, 5-6,8**  **Line no:13-16, 17, 33, 113-118, 171-177** |
|  | **Short title:** Rephrase and make it shorter | We are grateful to the reviewer for the suggestion. We have rephrased the short title in the title page of the revised manuscript. | **Title page** |
|  | **Introduction**  Line 115-122: repetitive words in both sentences  merge and make one sentence | We thank the reviewer for the suggestion. We have rephrased the text in the revised manuscript. | **Page no:5-6**  **Line no:112-116** |
|  | **Methods**  The intervention should be cited in the methodology section | We thank the reviewer for the comment. We have added brief text and cited the intervention development methodology (published elsewhere) in the methods section of the revised manuscript. | **Page no:8**  **Line no:168-174** |
| The authors feel that the manuscript has been immensely improved after incorporating the comments of the editorial board, and we thank the board for their time and efforts. | | | |
